# Mudanças em Nossos Tempos e nas Metas de Colesterol

**DOI:** 10.36660/abc.20200670

**Published:** 2020-09-18

**Authors:** Tania Leme da Rocha Martinez

**Affiliations:** 1 Hospital do Coração São Paulo SP Brasil Hospital do Coração (HCor), São Paulo, SP – Brasil

**Keywords:** Doenças Cardiovasculares/prevenção e controle, Fatores de Risco, LDL-Colesterol, Inibidores de Hidroximetilglutaril-CoA Redutases

Desde os anos 80, tem havido muitas abordagens quanto à interpretação das concentrações de colesterol na prática clínica. Houve até encontros médicos com títulos como – “Colesterol: Mito ou Verdade?”.

Ao mesmo tempo, ensaios para precipitação de HDL e cálculo do colesterol LDL utilizando a fórmula de Friedewald tornaram-se disponíveis nos laboratórios.

Estudos como o PROCAM e o *Framingham Heart Study* foram convincentes sobre sua importância e também sobre o risco e a proteção associados ao colesterol LDL e HDL, respectivamente.

Ensaios foram realizados ao longo de todos esses anos e várias Diretrizes e Documentos de Consenso foram apresentados e atualizados periodicamente em muitos países e em todos os continentes.

O colesterol como fator de risco é definido com mais precisão pelo LDL-colesterol e não-HDL-colesterol, e o valor alvo para cada indivíduo é definido pelos Escores de Risco que levam em consideração todos os fatores de risco.

Atualmente, estamos enfrentamos diferenças ao comparar as Diretrizes Brasileiras com as norte-americanas e até mesmo com as europeias.

Esta edição, o artigo de Cesena et al.^1^ compara nossas Diretrizes mais recentes às norte-americanas, no que diz respeito principalmente à indicação para o uso de estatinas.

O mesmo grupo de autores fez o mesmo com as diretrizes anteriores em 2017 para ambas.^2^

Nos dois artigos, as comparações apontam para o fato de que as Diretrizes Brasileiras são mais propensas a indicar a prescrição de estatinas do que as diretrizes norte-americanas, considerando a mesma estratificação de risco.

A abordagem brasileira apresenta mais semelhanças com a diretriz europeia, e é muito provável que o motivo dessas diferenças seja muito semelhante ao apontado em uma revisão recente^3^ que comparou as diretrizes da AHA às da ESC. Além de comparar os critérios em cada uma das diretrizes, os autores mencionam que, “Uma das principais razões para essas diferenças é a incorporação de considerações de custos pelas diretrizes da AHA-ACC, enquanto as diretrizes da ESC-EAS consideram um cenário ideal com recursos ilimitados”.^4-7^

A principal mensagem de todas as diretrizes é levar em consideração seu escopo e individualizá-lo para cada paciente em particular.

Considerando as semelhanças de finalidade das diretrizes, na realidade existem lacunas nas crenças e práticas no manejo da dislipidemia em diferentes países, muito bem documentadas em uma pesquisa médica na Web,^8^ e levando-se em consideração a globalização e os recursos da Web, essas diferenças serão melhor modeladas de acordo com as características regionais.

Por último, mas não menos importante, não nos esqueçamos do lembrete de uma ação global em relação à conscientização para os casos de Hipercolesterolemia Familiar (HF), reduzindo assim a carga clínica e pública desta apresentação. Essa doença subdiagnosticada e sub-tratada leva a morbidade e mortalidade prematuras devido a doença cardiovascular aterosclerótica^9^

Para facilitar as tarefas de Cálculo da Estratificação de Risco e da previsão de HF, há dois aplicativos independentes na seção do Departamento de Aterosclerose do site da Sociedade Brasileira de Cardiologia. Quanto maior a precisão na definição do valor alvo do colesterol LDL a ser alcançado, mais eficaz será a prática clínica.
