# Supplementary material for: Mudanças em Nossos Tempos e nas Metas de Colesterol
Source: Arq Bras Cardiol. 2020 Sep 18;115(3):450–1. [Article in Portuguese] doi: 10.36660/abc.20200670 (PMC9363088; doi:10.36660/abc.20200670)
Supplement: Translation

# Translation: Changes in Our Times and in Cholesterol Targets

**Authors:** Tania Leme da Rocha Martinez

**Affiliations:** 1 Brazil Hospital do Coração (HCor), São Paulo, SP – Brazil

**Keywords:** Cardiovascular Diseases/prevention and control, Risk Factors, Cholesterol-LDL, Hydroxymethylglutaryl- CoA Reductase Inhibitors

Since the 80s, many approaches have emerged regarding the interpretation of cholesterol levels in clinical practice.

There have even been medical meetings with titles such as: “Cholesterol: Myth or Truth”?

At that same time, HDL precipitation assays and LDL-cholesterol calculation using the Friedewald Formula became available in the laboratories.

Studies such as the PROCAM and The Framingham Heart Study were convincing regarding their importance and also of the risk and protection associated with LDL and HDL cholesterol, respectively.

Trials have been performed throughout all these years and several Guidelines and Consensus Documents have been presented and updated from time to time in many countries and in all continents.

Cholesterol as a risk factor is more accurately defined by both LDL and Non-HDL Cholesterol and the target value for each individual is defined by the Risk Scores that take all the risk factors into consideration.

At present, we are currently facing differences when comparing Brazilian Guidelines to the American and even to the European ones.

In this issue, the article of the Cesena et al.^1^ compares our most recent Guidelines to the North American ones, regarding mainly the indication for statin use.

The same group of authors had done the same with the former in 2017 for both.^1^

In both articles, the comparisons show that the Brazilian Guidelines are more prone to indicate the prescription of statins than the North American ones, considering the same risk stratification.

The Brazilian approach shows more similarities to the European guideline and it is very likely that the reason for the differences is very similar to the ones pointed out in a recent review^2^ that compared the AHA to the ESC guidelines. Apart from comparing the criteria in each of them, the authors mention that “One of the main reasons for these differences is the incorporation of cost value considerations by the AHA-ACC guidelines, whereas the ESC-EAS guidelines consider an ideal setting with unlimited resources”.^3-6^

The main message from all of the guidelines is to take its scope into consideration and individualize it for each particular patient.

Considering the similarities of purpose of the guidelines, in reality there are gaps in beliefs and practice regarding the management of dyslipidemia in different countries, which has been very well documented in a web-based physician survey^7^ and taking into account globalization and web resources, these differences will be better shaped according to regional characteristics.

Last but not least, let us not forget the reminder for a global action regarding awareness for cases of Familial Hypercholesterolemia (FH), thus reducing the clinical and public burden of this presentation. This underdiagnosed and undertreated disease leads to premature morbidity and mortality due to atherosclerotic cardiovascular disease.^8^

In order to facilitate the tasks of Risk Stratification Calculation and of FH presumption there are two independent Apps in the Atherosclerosis Department section of the Brazilian Society of Cardiology site. The more accurate the setting of the goal of the LDL-cholesterol level to be achieved, the more effective the clinical practice will be.
